# Influence of Wind Pressure on the Carbonation of Concrete

**DOI:** 10.3390/ma8084652

**Published:** 2015-07-24

**Authors:** Dujian Zou, Tiejun Liu, Chengcheng Du, Jun Teng

**Affiliations:** Shenzhen Graduate School, Harbin Institute of Technology, Shenzhen 518055, China; E-Mails: zoudujian@163.com (D.Z.); duchengcheng0410@gamil.com (C.D.); tengj@hit.edu.cn (J.T.)

**Keywords:** concrete, carbonation, penetration mass transfer, wind pressure, Klinkenberg effect

## Abstract

Carbonation is one of the major deteriorations that accelerate steel corrosion in reinforced concrete structures. Many mathematical/numerical models of the carbonation process, primarily diffusion-reaction models, have been established to predict the carbonation depth. However, the mass transfer of carbon dioxide in porous concrete includes molecular diffusion and convection mass transfer. In particular, the convection mass transfer induced by pressure difference is called penetration mass transfer. This paper presents the influence of penetration mass transfer on the carbonation. A penetration-reaction carbonation model was constructed and validated by accelerated test results under high pressure. Then the characteristics of wind pressure on the carbonation were investigated through finite element analysis considering steady and fluctuating wind flows. The results indicate that the wind pressure on the surface of concrete buildings results in deeper carbonation depth than that just considering the diffusion of carbon dioxide. In addition, the influence of wind pressure on carbonation tends to increase significantly with carbonation depth.

## 1. Introduction

Concrete material has been used as a major construction material for over one hundred years. It becomes increasingly important for the durability of reinforcement concrete (RC) structures. Both European and Italian Standards have recently renewed their attention on the durability of RC constructions [1,2,3,4,5]. The mechanical properties of reinforced concrete can be deteriorated due to chemical, physical and environmental attacks. Among the most frequent environmental attacks affecting RC structures, carbonation is one of the major factors to cause deterioration and is manifested by lowering the pH of concrete pore solutions from 12.6 to less than 9. This will lead to destroying the steel passive oxide film. The reinforcement corrosion induced by carbonation has been considered a major cause of deterioration of RC structures. To prevent premature deterioration of concrete structures, research is currently conducted to understand the physical and chemical mechanisms of deterioration through experimental studies and numerical simulations [6,7,8,9]. The influence of heat, moisture, coatings, carbon dioxide concentration and admixtures on carbonation process has been studied and corresponding coupled models were constructed [10,11,12,13,14,15,16,17,18].

In the case of carbonation, carbon dioxide reacts with constituents of hydrated cement and the most significant reaction is that carbon dioxide reacts with calcium hydroxide. As a consequence, the basic factor influencing carbonation is the mass transfer of carbon dioxide into the hardened cement paste. It is generally accepted that the carbonation rate is controlled by the diffusion of carbon dioxide into concrete pore system with a concentration difference acting as driving force. Many diffusion-reaction carbonation models have been established recently [10,14,15,16]. However, it should be noted that the mass transfer of gas flow in porous material is not determined by diffusion alone. Convection mass transfer may be distinct in some situations, especially for the constructions constructed in flat terrains with frequent strong winds. According to the Гусейнов’s investigation on the carbonation depth of RC power transmission tower in Baku, the carbonation depth of tower’s windward side and leeward side was 1.5 to 2 times larger than the other sides when the towers exposed to strong wind in the long term [19]. Qu *et al.* conducted two accelerated experimental investigations on rectangular and T-type concrete beams in wind tunnel. The high concentration (15% ± 2%) CO_2_ was used and the wind velocity was 5.5 m/s. It is concluded that the wind pressure and swirl have a significant impact on concrete carbonation [20]. It should be noted that the majority of available documents concerning the carbonation of concrete did not take convection mass transfer into consideration. This study investigates the influence of wind pressure on the carbonation of concrete. The carbon dioxide penetration process was analyzed based on Darcy’s law and then a penetration-reaction carbonation model was presented. Finally, the influence of steady wind flow and fluctuating wind flow on carbonation was studied.

## 2. Mass Transfer of Carbon Dioxide in Concrete

**Figure 1:**
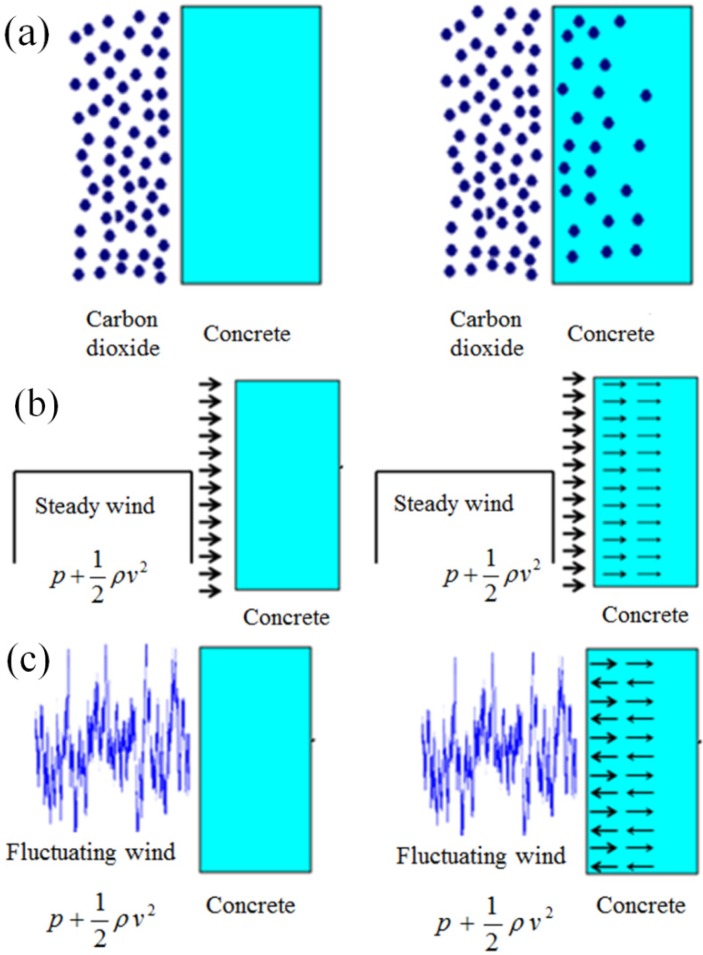
Mass transfer of carbon dioxide in concrete: (**a**) diffusion; (**b**) penetration induced by steady wind flow; and (**c**) penetration induced by fluctuating wind flow.

Concrete material is a typical porous media. The mass transfer in porous media includes two methods. One is the molecular diffusion induced by random motion of molecules in fluid. Another one is the convection mass transfer induced by macroscopic motion of fluid. The macroscopic motion of fluid in porous media is determined by capillary attraction, pressure, gravity, *etc.* In particular, the mass transfer induced by pressure difference is called penetration mass transfer. In the case of mass transfer of carbon dioxide in porous concrete, under wind pressure, molecular diffusion and penetration mass transfer of carbon dioxide coexist. As shown in Figure 1a, in proper conditions, the concentration difference makes carbon dioxide diffuse into concrete and the diffusion will be continuous until the reactants (such as calcium hydroxide) are used up. As shown in Figure 1b,c, when concrete is under wind pressure, carbon dioxide can penetrate into the concrete. There are also some differences for penetration between steady wind flow and fluctuating wind flow. Mass transfer of carbon dioxide in concrete induced by fluctuating wind flow is more effective than steady wind flow. The rapid variation of wind pressure results in a greater mass transfer speed through the gas flow transfers into and out of the concrete. As a consequence, the carbonation depth can be affected by the wind pressure on concrete surface.

### 2.1. Carbon Dioxide Diffusion Process

The process of carbon dioxide diffusion into concrete material can be described by Fick’s law, as in Equation (1). Taking the loss of carbon dioxide reaction with calcium hydroxide into consideration, the differential Equation (1) can be rewritten as Equation (2).

(1) $$\frac{\partial C_{{\text{co}}_{\text{2}}}}{\partial t}=\nabla \cdot (D_{{\text{co}}_{\text{2}}}\nabla (C_{{\text{co}}_{\text{2}}}))$$

(2) $$\frac{\partial C_{{\text{co}}_{\text{2}}}}{\partial t}=\nabla \cdot (D_{{\text{co}}_{\text{2}}}\nabla (C_{{\text{co}}_{\text{2}}}))-RC_{{\text{co}}_{\text{2}}}C_{{\text{Ca(OH}}_{\text{2}}\text{)}}$$

where $C_{{\text{co}}_{\text{2}}}$is the concentration of carbon dioxide, $C_{{\text{Ca(OH)}}_{\text{2}}}$is the concentration of calcium hydroxide (mol/L^−1^), $D_{{\text{co}}_{\text{2}}}$is the diffusion coefficient of carbon dioxide (m^2^/s), *R* is the rate constant of the reaction, and *t* is the time (s).

### 2.2. Carbon Dioxide Penetration Process

The process of carbon dioxide penetration into concrete material can be described by Darcy’s law [21]. When the gas flow is close to the ground surface, the variation of atmospheric pressure is negligible. The gas flow can be accepted as incompressible flow. Therefore, the macro flow velocity can be described as Equation (3). For the horizontal gas flow, the effect of gas gravity on macroscopic motion of flow is negligible. The macro flow velocity can be rewritten by Equation (4).

(3) $$j_{f}=-\frac{k}{\text{μ}}(\nabla p-\text{ρ}g)$$

(4) $$j_{f}=-\frac{k}{\text{μ}}\nabla p$$

where *j_f_* is the transmitted flux (m/s), *k* is the gas permeability (m^2^), μ is the viscosity coefficient (Pa·s), *p* is the wind pressure (Pa), ρ is the density of gas (g/cm^3^), and *g* is the gravity vector (m/s^2^).

#### 2.2.1. Governing Equations of Penetration-Reaction

**Figure 2:**
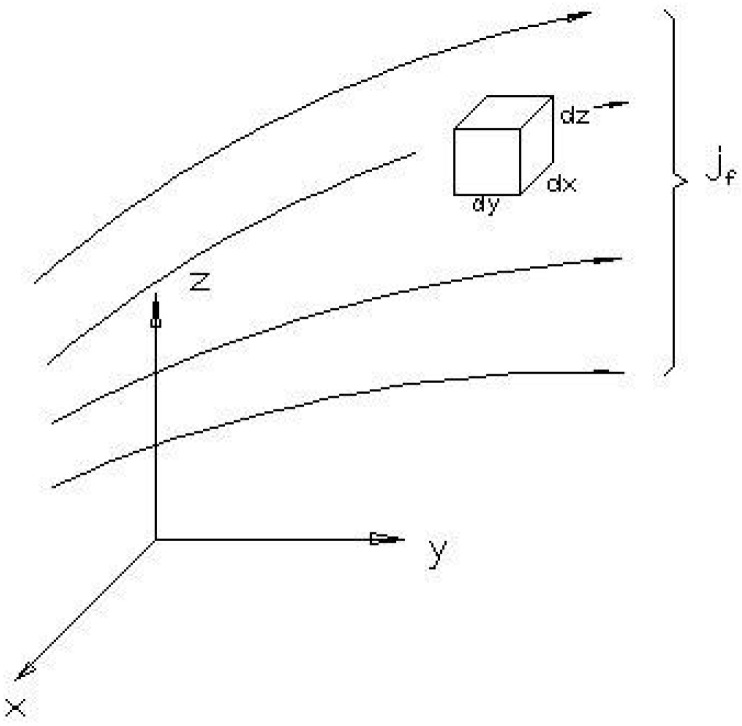
A unit volume in Cartesian coordinate system.

For a unit volume shown in Figure 2, the mass conservation can be described as Equation (5),

(5) $$\frac{\partial {\text{ρ}}_{{\text{co}}_{\text{2}}}}{\partial t}+\frac{\partial ({\text{ρ}}_{{\text{co}}_{\text{2}}}j_{f,x})}{\partial x}+\frac{\partial ({\text{ρ}}_{{\text{co}}_{\text{2}}}j_{f,y})}{\partial y}+\frac{\partial ({\text{ρ}}_{{\text{co}}_{\text{2}}}j_{f,z})}{\partial z}=0$$

where ${\text{ρ}}_{{\text{co}}_{\text{2}}}$ is the density of carbon dioxide (g/cm^3^), *j_f,x_* is the transmitted flux in *x*-direction (m/s), *j_f,y_* is the transmitted flux in *y*-direction (m/s), and *j_f,z_* is the transmitted flux in *z*-direction (m/s). Equation (5) can be simplified to

(6) $$\frac{\partial {\text{ρ}}_{{\text{co}}_{\text{2}}}}{\partial t}+\nabla \cdot ({\text{ρ}}_{{\text{co}}_{\text{2}}}j_{f})=0$$

Substituting Equation (4) into Equation (6),

(7) $$\frac{\partial {\text{ρ}}_{{\text{co}}_{\text{2}}}}{\partial t}-\nabla \cdot (\frac{k}{\mu }{\text{ρ}}_{{\text{co}}_{\text{2}}}\nabla p)=0$$

Transposition of terms,

(8) $$\frac{\partial {\text{ρ}}_{{\text{co}}_{\text{2}}}}{\partial t}=\nabla \cdot (\frac{k}{\text{μ}}{\text{ρ}}_{{\text{co}}_{\text{2}}}\nabla p)$$

Taking the loss of carbon dioxide reaction with calcium hydroxide into consideration, the differential Equation (8) can be replaced by Equation (9),

(9) $$\frac{\partial {\text{ρ}}_{{\text{co}}_{\text{2}}}}{\partial t}=\nabla \cdot (\frac{k}{\text{μ}}{\text{ρ}}_{{\text{co}}_{\text{2}}}\nabla p)-R{\text{ρ}}_{{\text{co}}_{\text{2}}}{\text{ρ}}_{{\text{Ca(OH}}_{\text{2}}\text{)}}$$

where ${\text{ρ}}_{{\text{Ca(OH)}}_{\text{2}}}$ is the density of calcium hydroxide (g/cm^3^). For mass transfer of carbon dioxide in porous concrete, the Equation (9) can be further replaced by Equation (10),

(10) $$\frac{\partial \phi {\text{ρ}}_{{\text{co}}_{\text{2}}}}{\partial t}=\nabla \cdot (\frac{k}{\text{μ}}{\text{ρ}}_{{\text{co}}_{\text{2}}}\nabla p)-R\phi {\text{ρ}}_{{\text{co}}_{\text{2}}}{\text{ρ}}_{{\text{Ca(OH}}_{\text{2}}\text{)}}$$

where Φ is the concrete porosity (%).

#### 2.2.2. Gas Flow Equation with Klinkenberg Effects

To evaluate the penetration-reaction carbonation, the most difficult step is to determine the gas flow in porous concrete. The ground surface system can be taken as isothermal, then ideal gas law is a better approximation to the near surface air flow. According to the mass transfer theory, under isothermal conditions, gas flow in concrete is governed by a mass balance equation [22],

(11) $$\frac{\partial (\phi \text{ρ})}{\partial t}+\nabla \cdot (\text{ρ}V)=0$$

where *V* is the Darcy’s velocity of the gas, defined as

(12) $$V=-\frac{k_{\text{g}}}{\text{μ}}(\nabla P-\text{ρ}g)$$

The ideal gas law here is used to describe the relation between pressure and gas density,

(13) ρ=βP

where β is the compressibility factor, defined as

(14) $$\text{β}=\frac{M_{\text{g}}}{ST}$$

where *M*_g_ is the molecular weight of the gas, *T* is the constant temperature (°C), *S* is the universal gas constant, and *k*_g_ is the effective gas permeability, defined as

(15) $$k_{\text{g}}=k_{\infty }(1+\frac{b}{p})$$

where *b* is the Klinkenberg coefficient (Pa), *k_∞_* is the absolute gas/water permeability (m^2^). 

When gravity effects are ignored, combing Equations (11)–(13), and (15) will give

(16) $$\nabla \cdot (\frac{k_{\infty }}{\text{μ}}(P+b)(\nabla P))=\phi \text{β}\frac{\partial P}{\partial t}$$

In terms of the new variable, *P*_b_=*P*+*b*, Equation (16) can be written as

(17) $$\nabla \cdot (\nabla {P_{\text{b}}}^{2})=\frac{1}{\alpha }\frac{\partial {P_{\text{b}}}^{2}}{\partial t}$$

The differential Equation (17) can be solved by Finite Element Method to determine the distribution of pressure in concrete, after the initial conditions and BC conditions are given. Then the gas flow in porous concrete is given and the penetration and consumption of carbon dioxide can be determined.

### 2.3. Penetration-Reaction Carbonation Model

Many mathematical/numerical carbonation models have been constructed by researchers [14,15,16]. To study the influence of pressure on carbonation, the governing differential equation representing for the diffusion and consumption of carbon dioxide in the concrete can be replaced by the penetration-reaction equation. Taking Park’s diffusion-reaction carbonation model as an example [16] (see Appendix: diffusion-reaction carbonation model), the penetration-reaction carbonation model can be shown as Equations (18)–(20). Equation (18) represents the penetration and consumption of carbon dioxide in the concrete, Equation (19) represents the diffusion and consumption of calcium hydroxide, and Equation (20) represents the generation of calcium carbonate. It should be noted that Ca(OH)_2_ and C–S–H in concrete are normally carbonated simultaneously [23,24]. C–S–H is not considered a reactant in this model for simplification.

(18) $$\frac{\partial \phi {\text{ρ}}_{{\text{co}}_{\text{2}}}}{\partial t}=\nabla \cdot (\frac{k}{\text{μ}}{\text{ρ}}_{{\text{co}}_{\text{2}}}\nabla p)-R\phi {\text{ρ}}_{{\text{co}}_{\text{2}}}{\text{ρ}}_{{\text{Ca(OH}}_{\text{2}}\text{)}}$$

(19) $$\frac{\partial {\text{ρ}}_{{\text{Ca(OH}}_{\text{2}}\text{)}}}{\partial t}=D_{{\text{Ca(OH)}}_{\text{2}}}\frac{\partial^{2}{\text{ρ}}_{{\text{Ca(OH}}_{\text{2}}\text{)}}}{\partial x^{2}}-R\phi {\text{ρ}}_{{\text{co}}_{\text{2}}}{\text{ρ}}_{{\text{Ca(OH}}_{\text{2}}\text{)}}$$

(20) $$\frac{\partial {\text{ρ}}_{{\text{CaCO}}_{\text{3}}}}{\partial t}=R\phi {\text{ρ}}_{{\text{co}}_{\text{2}}}{\text{ρ}}_{{\text{Ca(OH}}_{\text{2}}\text{)}}$$

where ${\text{ρ}}_{{\text{CaCO}}_{\text{3}}}$is the density of calcium carbonate.

The validity of this proposed penetration-reaction carbonation model should be verified before the influence of wind pressure on concrete carbonation is discussed. However, there are few carbonation models considering the influence of pressure on concrete carbonation. So it is hard to validate the proposed penetration-reaction carbonation model using existing theoretical and numerical models. With regard to experiments, some research has been conducted though experimental investigations in order to develop a carbon dioxide mitigation technology [25,26]. The concrete is used to store carbon dioxide under high CO_2_ pressure. Nevertheless, the published literature mostly focuses on the ability of carbon dioxide mitigation. The carbonation depth in accelerated carbonation tests was nearly neglected. Atis carried out an accelerated carbonation experiment in a controlled environment, which was used to assess the carbonation of fly ash concrete [27]. In this verification, his experimental results were used to validate the proposed penetration-reaction carbonation model. The parameters of controlled environment are shown in Table 1. The proportion of cement:water:stone:sand used in the test was 1:0.55:3:1.5. The 28-day cubic compressive strength of concrete was 51.20 MPa. Concrete specimens with different curing times were all exposed in the control environment for two weeks. Further details of this test can be found in Reference [27].

**Table 1:** Parameters of controlled environment.

Temperature	Concentration of CO_2_	Relative Humidity	Pressure
20 °C	4.70%	65%	1 bar above ambient

Permeability is an intrinsic characteristic of concrete, which should be independent of the fluid and only dependent on the pore structure of concrete. However, the published experiments show that there is a difference between gas and water permeability for concrete. The relationship between gas and water permeability coefficients (*k*_g_ and *k_∞_*) is given in Equation (15), where *b* is a constant for a given porous media and a given gas. Equation (21) was derived by Bamforth to calculate the value of *b* for concrete [28].

(21) $$b=1.635\times 10^{-8}k_{\infty }^{-0.5227}$$

In the case in point, given the water/cement ratio (0.55) and cubic compressive strength (51.20 MPa), the water permeability was determined as 10^−12.72^ m/s [29], and then the gas permeability was calculated by Equation (15). In the meantime, the influence of curing time on permeability of the concrete was considered. 

Viscosity coefficient of gas depends mostly on the temperature, and it can be calculated by Sutherland’s formula:

(22) $$\text{μ}={\text{μ}}_{0}\times (\frac{0.555T_{0}+C}{0.555T+C})\times {\left(T/T_{0}\right)}^{1.5}$$

where μ is the viscosity coefficient at temperature *T*, μ_0_ is the reference viscosity coefficient at temperature *T*_0_, and *C* is the Sutherland’s constant (120 for standard air, 240 for carbon dioxide). At 20 °C in the control environment, the value of the viscosity coefficient was set as 1.48 × 10^−5^ Pa·s.

The amount of generated Ca(OH)_2_ in concrete depends on the cement hydration, and in general, the amount of Ca(OH)_2_ is about 30% of cement mass when the cement hydration completes. Consequently, the mass concentration of Ca(OH)_2_ can be described as follows:

(23) $${\text{ρ}}_{{\text{Ca(OH}}_{\text{2}}\text{)}}\text{=}0.3\cdot \text{Q}\cdot \text{α}$$

where *Q* is the density of cement before hydration, and α is the hydration ratio. In this case study, the area of full carbonation was assumed to be the positions at which the quantity of Ca(OH)_2_ was reduced to half of its initial value [16].

It has been validated by Hukujima [30] and Saeki [31] that the key factor affecting the diffusion of Ca^2+^ is the moisture in concrete. Furthermore, Hukujima accepted that the diffusion coefficient of Ca^2+^ conforms to exponential functions of the water content in concrete. The actual chemical reaction between CO_2_ and Ca(OH)_2_ is complex. In this study, the reaction between CO_2_ and Ca(OH)_2_ was considered as a first-order reaction. The reaction rate constant is normally affected by temperature, concentration, pressure, *etc.* In most situations, the reaction rate constant of a special chemical reaction is usually measured through an experiment. So far, few literatures have specified the reaction rate constant of carbonation clearly. Thus, it is very hard to make sure of the exact value of the reaction rate constant in an accelerated carbonation test under high pressure. In this case study, the diffusion coefficient of Ca^2+^ and reaction constant rate were assumed as 1 × 10^−12^ m^2^/s and 5 × 10^−5^ m^3^/mol/s, respectively [16].

The concrete was carbonized in a control environment under one bar (0.1MPa) pressure above ambient, which is far greater than wind pressure. Therefore, the travel of CO_2_ into concrete from outside mostly depends on the penetration mass transfer, and the mass transfer of CO_2_ induced by diffusion can be neglected. The estimated carbonation depth by the proposed model is shown in Table 2. It is observed that the consistency between test and simulation results is not very well. All calculated carbonation depths are bigger than measured carbonation depths. The reason why the test results were over-estimated, is due to the generated redundant water by carbonation reaction. For every mole of CaCO_3_ generated by the reaction, a mole of water was generated. In the process of accelerated carbonation, a mass of CaCO_3_ was generated accompanying with the same mole of water in a short time (2 weeks). The generated water leads to an evident increase in the relative humidity in concrete, which decreases the transfer efficiency of CO_2_ permeated into the internal concrete. Furthermore, the greater the amount of water produced in a period of time, the less CO_2_ penetrates into concrete in the same time range. It indicates that the greater the carbonation depth in a period of time is, the larger the calculation error occurs, when the effect of generated water on penetration mass transfer of CO_2_ is neglected. However, it is hard to quantitatively analyze the influence of generated water on the adverse effect of CO_2_ travel in concrete. To improve the accuracy of this proposed model, more experiments are needed for further calibration of the adverse effect, caused by evaporation and sorption of water in concrete, on mass transfer of CO_2_.

**Table 2:** Carbonation depth of concrete in test and simulation.

	Curing Time (at 100% Relative Humidity with 20 °C)			
	3 days	7 days	28 days	3 months
Test results (mm)	9.10	7.40	4.50	3.30
Simulation (mm)	11.01	8.49	4.97	3.52

## 3. Effect of Wind Pressure on Carbonation

When the concrete is under wind pressure, the diffusion-reaction and penetration-reaction coexist in concrete. A comparative analysis can be done to evaluate the influence of wind pressure on the carbonation. Since it is too complex to consider the diffusion and penetration together, we first only studied the carbonation induced by diffusion ignoring the effect of wind pressure and then we evaluated the carbonation induced by penetration. Finally, the diffusion-induced carbonation and penetration-induced carbonation were compared. In most situations, the diffusion-induced carbonation rate of concrete decreases over time, then the influence of wind flow on carbonation is time dependent compared with diffusion-induced carbonation. In general, the cover thickness of concrete buildings is around 30 mm. For studying the diffusion-induced carbonation and penetration-induced carbonation in total working life of structures, seven initial conditions were considered, in which the initial full carbonation depths were assumed to be 0 mm, 5 mm, 10 mm, 15 mm, 20 mm, 25 mm, and 30 mm, respectively.

### 3.1. Initial Conditions and Boundary Conditions

(1) For diffusion-induced carbonation (diffusion-reaction carbonation model showing in Appendix)

At *t* = 0, when *d* (depth of full carbonization) is 0 mm,

(24) $$x>0,\;C_{{\text{co}}_{\text{2}}}=0,\;C_{{\text{Ca(OH}}_{\text{2}}\text{)}}=C_{{\text{Ca(OH}}_{\text{2}}\text{)-Init}}$$

when *d* are 5 mm, 10 mm, 15 mm, 20 mm, 25 mm, and 30 mm,

(25) $$x>d,\;C_{{\text{co}}_{\text{2}}}=0,\;C_{{\text{Ca(OH}}_{\text{2}}\text{)}}=C_{{\text{Ca(OH}}_{\text{2}}\text{)-Init}}$$

(26) $$0<x\leq d,\;C_{{\text{co}}_{\text{2}}}=0,\;C_{{\text{Ca(OH}}_{\text{2}}\text{)}}=0$$

Equations (24)–(26) represent the initial conditions of concrete. The diffusion coefficient of carbon dioxide in concrete is affected by porosity and relative humidity. In the following case study, the diffusion coefficient was taken as a constant.

At *t* > 0,

(27) $$x=0:\;C_{co_{2}}=\phi \cdot C_{co_{2}-init}\;C_{Ca(OH_{2})}=0$$

(28) $$x\to +\infty :\;C_{Ca{\left(OH\right)}_{2}}=C_{Ca{\left(OH\right)}_{2}-init},\;C_{CO_{2}}=0$$

Equations (27) and (28) represent the initial conditions and boundary conditions for carbonation. Equation (27) is the carbon dioxide concentration on the surface of concrete.

(2) For penetration-induced carbonation

At *t* = 0, when *d* is 0 mm,

(29) $$x>0,\;{\text{ρ}}_{{\text{co}}_{\text{2}}}=0,\;{\text{ρ}}_{{\text{Ca(OH}}_{\text{2}}\text{)}}={\text{ρ}}_{{\text{Ca(OH}}_{\text{2}}\text{)-Init}},\;p=p_{0}$$

when *d* are 5 mm, 10 mm, 15 mm, 20 mm, 25 mm, and 30 mm,

(30) $$x>d,\;{\text{ρ}}_{{\text{co}}_{\text{2}}}=0,\;{\text{ρ}}_{{\text{Ca(OH}}_{\text{2}}\text{)}}={\text{ρ}}_{{\text{Ca(OH}}_{\text{2}}\text{)-Init}},\;p=p_{0}$$

(31) $$0<x\leq d,\;{\text{ρ}}_{{\text{co}}_{\text{2}}}=0,\;{\text{ρ}}_{{\text{Ca(OH}}_{\text{2}}\text{)}}=0,\;p=p_{0}$$

Equations (29)–(31) represent the initial conditions of concrete. The initial wind pressure on surface of concrete was considered as atmospheric pressure.

At *t* > 0,

(32) $$x=0:\;{\text{ρ}}_{{\text{co}}_{\text{2}}}=\phi \cdot {\text{ρ}}_{{\text{co}}_{\text{2}}\text{-init}}\;{\text{ρ}}_{{\text{Ca(OH}}_{\text{2}}\text{)}}=0,\;p=p_{0}+\frac{v^{2}}{1600}$$

(33) $$x\to +\infty :\;{\text{ρ}}_{{\text{Ca(OH)}}_{\text{2}}}={\text{ρ}}_{{\text{Ca(OH)}}_{\text{2}}\text{-init}},\;{\text{ρ}}_{{\text{CO}}_{\text{2}}}=0,\;p=p_{0}$$

Equations (32) and (33) represent the initial conditions and boundary conditions of penetration-induced carbonation. Equation (32) was applied to the wind pressure on the surface of concrete.

### 3.2. Numerical Parameters for Concrete and Wind Flow

The numerical parameters for the concrete used in this simulation were referred to the parameters in Saetta’s case study [13]. Table 3 shows the major characteristic parameters of the concrete and environmental conditions. In practical conditions, the diffusion coefficient is affected by the porosity and relative humidity. It is decreased by an increase in carbonation or relative humidity. With regard to the reaction rate constant, it depends on the temperature. For this numerical analysis, which focused on the influence of wind pressure on carbonation, the diffusion coefficient of carbon dioxide and reaction rate constant were considered as constants.

**Table 3:** Numerical parameters for the concrete and reaction.

*w*/c	Φ (%)	*T* (°C)	*C*_co2_ (%)	*D*_co2_ (m^2^/s)	*R* (m^3^/mol/s)	*k*_∞_ (m^2^)	μ (Pa·s)
0.38	15.24	20 °C	0.015	1 × 10^−8^	5 × 10^−5^	5.59 × 10^−17^	1.84 × 10^−5^

**Figure 3:**
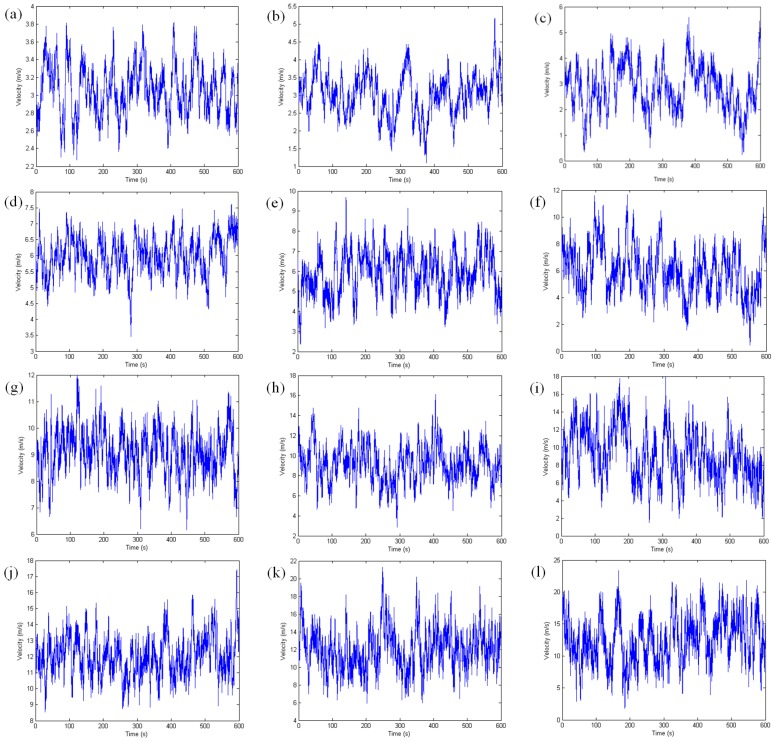

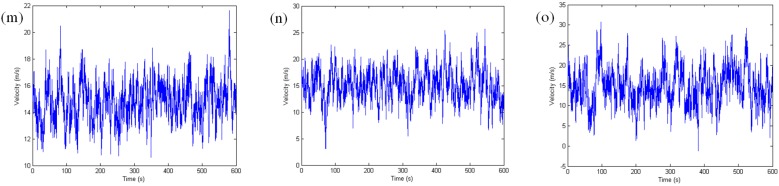
The time–history curves of wind flows with different turbulence intensities (MWV: mean wind velocity, TI: turbulence intensity): (**a**) MWV = 3 m/s, TI = 0.1; (**b**) MWV = 3 m/s, TI = 0.2; (**c**) MWV = 3 m/s, TI = 0.3; (**d**) MWV = 6 m/s, TI = 0.1; (**e**) MWV = 6 m/s, TI = 0.2; (**f**) MWV = 6 m/s, TI = 0.3; (**g**) MWV = 9 m/s, TI = 0.1; (**h**) MWV = 9 m/s, TI = 0.2; (**i**) MWV = 9 m/s, TI = 0.3; (**j**) MWV = 12 m/s, TI = 0.1; (**k**) MWV = 12 m/s, TI = 0.2; (**l**) MWV = 12 m/s, TI = 0.3; (**m**) MWV = 15 m/s, TI = 0.1; (**n**) MWV = 15 m/s, TI = 0.2; and (**o**) MWV = 15 m/s, TI = 0.3.

To study the influence of wind flow on the carbonation of concrete, the wind flows were simulated by Auto Regression model [32]. The mean wind velocities were supposed to be 3, 6, 9, 12 and 15 m/s, respectively, and the turbulence intensities were supposed to be 0, 0.1, 0.2 and 0.3, respectively. When the turbulence intensity was 0, it was steady wind flows. The time–history curves of wind flows with different turbulence intensities (0.1, 0.2 and 0.3) are shown in Figure 3.

### 3.3. Results of Numerical Analysis

**Figure 4:**
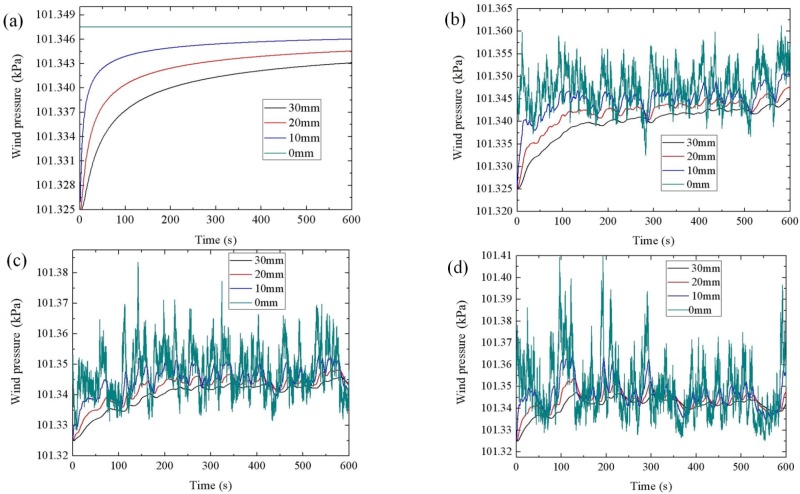
The time-history of wind pressure at various depths (MWV: mean wind velocity, TI: turbulence intensity): (**a**) MWV = 6 m/s, TI = 0; (**b**) MWV = 6 m/s, TI = 0.1; (**c**) MWV = 6 m/s, TI = 0.2; and (**d**) MWV = 6 m/s, TI = 0.3.

Figure 4 shows the time–history curves of wind pressure at various depths with a 6 m/s wind flow. It can be seen that higher fluctuating wind induces greater fluctuating wind pressure in internal concrete and the wind pressure of different depths have hysteresis effect. After the distribution of pressure is determined, as in the following figures, the penetration and consumption of carbon dioxide in the concrete can be evaluated.

**Figure 5:**
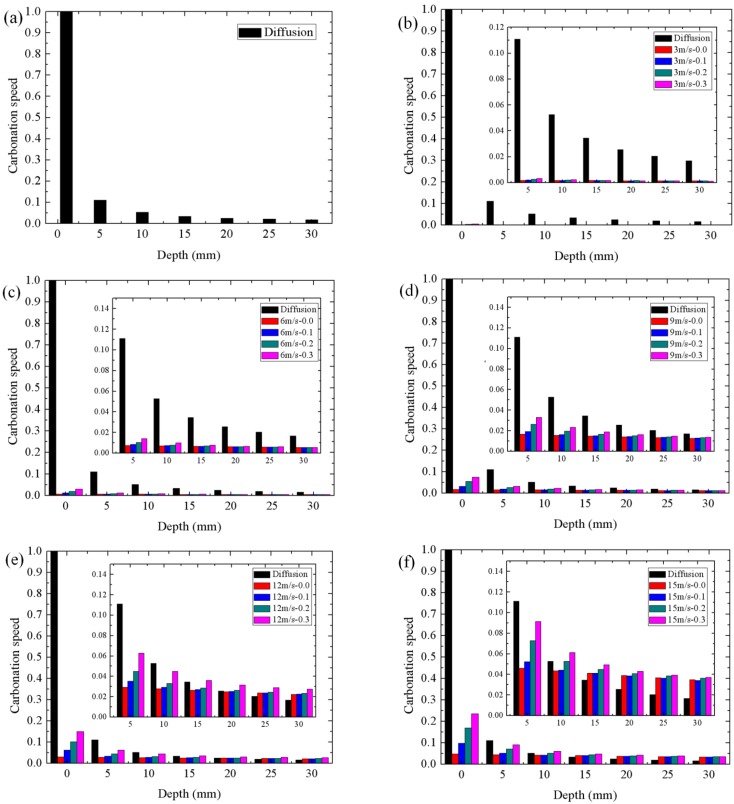
Comparison of carbonation speed at various depths induced by diffusion and penetration. (**a**) diffusion; (**b**) penetration at 3 m/s wind flow; (**c**) penetration at 6 m/s wind flow; (**d**) penetration at 9 m/s wind flow; (**e**) penetration at 12 m/s wind flow; and (**f**) penetration at 15 m/s wind flow.

Figure 5 illustrates the comparison of carbonation speed induced by diffusion and penetration. The *x*-axis corresponds to the depths of full carbonation, which are the initial conditions to further carbonation. The *y*-axis corresponds to the carbonation speed, which is defined as the ratio that the absorptive amount of carbon dioxide in ten minutes at different initial conditions divided by the absorptive amount of carbon dioxide in ten minutes induced by diffusion when the initial full carbonation depth is 0 mm. As shown in Figure 5a, the carbonation speed induced by diffusion decreased significantly with the increasing depth of full carbonation. This is consistent with the known square-root law, which describes the relationship between carbonation depth and time.

As shown in Figure 5b–f, higher mean wind velocity can induce greater penetration carbonation. When mean wind velocity is around 3m/s even with high turbulence intensity, the influence of wind pressure on carbonation is negligible. It is obvious that the diffusion-induced carbonation model is applicable in total carbonation process when the wind velocity is less than 3 m/s. However, when the mean wind velocity is over 6 m/s, the influence of wind pressure on carbonation is becoming significant. If the concrete structures suffer long-term strong wind, the carbonation induced by wind pressure should be considered. For certain wind velocity, the greater turbulence intensity can induce greater penetration-induced carbonation. However, when the carbonation depth is larger than 30 mm, the influence of turbulence intensity on carbonation becomes slightly significant.

It should be noted that, at the initial carbonation stage, the influence of wind pressure on carbonation is negligible. Even when mean wind velocity is 12 m/s and turbulence intensity is 0.3, the carbonation speed induced by wind flow is only about 15% of carbonation speed induced by diffusion. The diffusion-induced carbonation is dominant. When the carbonation depth is larger than 5 mm, the influence of wind pressure on carbonation tends to be significant. It depends on the significant decrease of carbonation speed induced by diffusion with the increasing carbonation depth. Under the long-term wind pressure, the carbonation induced by wind pressure became significant with time. When the carbonation depth is greater than 20 mm, under the mean wind velocity 12 m/s, the carbonation speed induced by wind pressure is higher than the carbonation speed induced by diffusion.

## 4. Conclusions

A penetration-reaction carbonation model was constructed that takes account of the penetration mass transfer of CO_2_ in concrete, and this model was validated by an accelerated test results. It is shown that this model can be used to estimate the carbonation depth of concrete under high pressure, and the validity of this model can be improved by calibrating the adverse effect induced by the redundant water generated in carbonation reaction.

For the wind flow, the higher mean velocity and turbulence intensity induce greater influence on carbonation. When the wind velocity is lower than 3m/s, the diffusion-reaction carbonation model is applicable to total carbonation process. At a particular wind velocity, higher turbulence intensity induces greater penetration carbonation. However, when the carbonation depth is larger than 30 mm, the influence of turbulence intensity on carbonation becomes slightly significant. 

At the initial carbonation stage, the influence of wind flow on carbonation is negligible. Even when the mean wind velocity is 12 m/s and turbulence intensity is 0.3, the carbonation speed induced by wind flow is only 15% of the carbonation speed induced by diffusion. When the carbonation depth is over 5 mm, the influence of wind flow on carbonation tends to be significant. Especially for the carbonation depth beyond 20 mm, at the mean wind velocity of 12 m/s, the carbonation speed induced by wind flow is higher than the carbonation speed induced by diffusion.

In this study, the influence of mass transfer induced by wind flow on concrete porosity is not taken into account. Therefore, the influence of wind flow on carbonation may be underestimated. The total carbonation depth *D*_total_ = *α* × (*D*_diffusion_ *+ D*_penetration_), this numerical analysis and the coefficient α still need to be experimentally verified. Nevertheless, this study validated the applicability of the proposed model in the area of carbonation depth estimation under high pressure. Furthermore, this study suggests that the carbonation depth increases with higher wind pressure when the penetration-induced carbonation and diffusion-induced carbonation coexist, and the influence of wind flow on carbonation tends significant with the increasing carbonation depth in the long term.

## Appendix

### Diffusion-Reaction Carbonation Model

$$\frac{\partial C_{{\text{co}}_{\text{2}}}(x,t)}{\partial t}=D_{{\text{co}}_{\text{2}}}\frac{\partial^{2}C_{{\text{co}}_{\text{2}}}(x,t)}{\partial x^{2}}-RC_{{\text{co}}_{\text{2}}}C_{{\text{Ca(OH}}_{\text{2}}\text{)}}$$

$$\frac{\partial C_{{\text{Ca(OH}}_{\text{2}}\text{)}}}{\partial t}=D_{{\text{Ca(OH)}}_{\text{2}}}\frac{\partial^{2}C_{{\text{Ca(OH}}_{\text{2}}\text{)}}}{\partial x^{2}}-RC_{{\text{co}}_{\text{2}}}C_{{\text{Ca(OH}}_{\text{2}}\text{)}}$$

$$\frac{\partial C_{{\text{CaCO}}_{\text{3}}}}{\partial t}=RC_{{\text{co}}_{\text{2}}}C_{{\text{Ca(OH}}_{\text{2}}\text{)}}$$

## Notation

$C_{{\text{co}}_{\text{2}}}$: concentration of carbon dioxide;
$C_{{\text{Ca(OH)}}_{\text{2}}}$: concentration of calcium hydroxide, mol/L*^−^*^1^;
$C_{{\text{CaCO}}_{\text{3}}}$: concentration of calcium carbonica, mol/L*^−^*^1^;
$D_{{\text{co}}_{\text{2}}}$: diffusion coefficient of carbon dioxide, m^2^/s;
$D_{{\text{Ca(OH)}}_{\text{2}}}$: diffusion coefficient of Ca^2+^, m^2^/s;
*R*: rate constant of the reaction,
*t*: time, s.
